# Essential thrombocythemia, unremarkable cause of atypical chest pain with simple yet effective treatment: a case report

**DOI:** 10.4076/1757-1626-2-7011

**Published:** 2009-09-11

**Authors:** Kevin J Patel, Amit Banga, Shahibzada U Latif

**Affiliations:** 1 Department of Medicine, Michigan State University, B301 MSU Clinical Center, 138 Service Road, East Lansing, MI 48824, USA

## Abstract

**Introduction:**

The cause of chest pain in patients presenting to the emergency room often remains unclear. We present a case of essential thrombocythemia as a novel cause of atypical chest pain, which responded dramatically to a simple treatment intervention.

**Case presentation:**

A 54-year-old patient presenting with atypical chest pain was found to have essential thrombocythemia as a cause for her chest pain. She responded dramatically to aspirin therapy and had no recurrence of symptoms over 3 months.

**Conclusion:**

Essential thrombocythemia should be considered as a differential cause in patient presenting with atypical chest pain, vasomotor symptoms and high platelet counts. These symptoms are generally more bothersome than dangerous and are usually controlled by low dose aspirin therapy.

## Introduction

Acute chest pain (CP) is one of the most common reasons for ER visits and its management involves establishing the cause while excluding potentially life-threatening conditions. It, therefore, poses a significant diagnostic challenge. Despite extensive work up to determine the cause of chest pain, the diagnosis often remains unclear leaving both the patient and the physician unsatisfied. We present a case of atypical chest pain due to a relatively unremarkable cause; Essential thrombocythemia (ET), which responded dramatically to a simple treatment intervention.

## Case presentation

A 54-year-old Caucasian female presented to ER with episodes of CP for the last 4 hours. The pain was characterised as acute onset, sharp, non-exertional, non-radiating pain localised to left side of the chest. It was 10/10 in severity with no discernable aggravating or relieving factors with each episode lasting for 2-3 minutes. Certain sensory symptoms had preceded the onset of CP. This comprised of sudden onset numbness of the left thumb followed by a tingling sensation in the shoulders with radiation distally into both hands. Subsequently, patient also developed severe, generalized, headache along with lights flashing across her eyes. By the time she reached the ER, her symptoms had completely resolved. Her past medical history was significant only for the diagnosis of essential thrombocythemia (ET) based on a bone marrow biopsy and a positive JAK2 mutation test. She had not required any previous treatment for this condition. Her baseline platelet count had ranged between 600 × 10^9^/l to 750 × 10^9^/l over the last one year. Initial laboratory investigations in the ER revealed normal basic metabolic panel and complete blood count except for a platelet count of 935 × 10^9^/l. Her chest pain was evaluated with three sets of cardiac enzymes and exercise stress echocardiogram which were all normal. Her chest X-ray did not reveal any infiltrates or pneumothorax. CT scan of chest with contrast showed no evidence of pulmonary embolism or aortic dissection. Her EKG revealed normal sinus rhythm. Her 24 hour continuous telemetry also did not show any arrhythmias. Her atypical, non-localizing, sensory symptoms with negative cardiopulmonary work up were subsequently attributed to vasomotor manifestations due to ET. The patient was reassured and started on a low dose aspirin therapy. At 12 weeks follow up, she reported no subsequent similar episodes.

## Discussion

Essential thrombocythemia (ET) is a clonal stem cell disorder characterized by a persistent, nonreactive thrombocythemic state that is not accounted for by any of the other chronic myeloproliferative disorders [1].

Our patient presented with classic vasomotor manifestations of ET, which may be present in approximately half of the patients with this myeloproliferative disorder. The common vasomotor symptoms experienced by these patients include headache, palpitations, atypical chest pain, distal paresthesias [2] or transient visual disturbances such as amaurosis fugax, scintillating scotomata and ophthalmic migraine [3]. Platelet-mediated ischemic and thrombotic processes in the end-arterial microvasculature are the likely cause for this symptoms [3]. The diagnosis of ET in our patient had been previously confirmed by appropriate tests. The vasomotor symptoms associated with ET are generally more distressing than dangerous, and are usually controlled by treatment with low dose of acetylsalicylic acid (ASA) [4]. Since our patient responded adequately to aspirin therapy, there was no need for cytoreductive intervention.

## Conclusion

Whereas the work up of acute chest pain to rule out potentially life threatening conditions is essential, the recognition and appropriate treatment of relatively unremarkable causes of chest pain like ET can relieve the patients' symptoms and may also reduce the excessive utilization of health care resources.

## Abbreviations

ASA: acetylsalicylic acid; CP: chest pain; CT: computer tomography; ER: emergency room; ET: essential thrombocythemia; EKG: electrocardiogram.

## Consent

Written informed consent was obtained from the patient for publication of this case report and accompanying images. A copy of the written consent is available for review by the Editor-in-Chief of this journal.

## Competing interests

The author(s) declare that they have no competing interests.

## Authors' contributions

KP assembled, analyzed and interpreted the patient data regarding the hematological disease. All authors contributed to writing the manuscript. All authors read and approved the final manuscript.
